# Molecular Recognition by Gold Nanoparticle-Based Receptors as Defined through Surface Morphology and Pockets Fingerprint

**DOI:** 10.1021/acs.jpclett.1c01365

**Published:** 2021-06-10

**Authors:** Laura Riccardi, Sergio Decherchi, Walter Rocchia, Giordano Zanoni, Andrea Cavalli, Fabrizio Mancin, Marco De Vivo

**Affiliations:** † Laboratory of Molecular Modeling & Drug Discovery, Fondazione Istituto Italiano di Tecnologia, Via Morego 30, 16163 Genova, Italy; ‡ Computational and Chemical Biology, Fondazione Istituto Italiano di Tecnologia, Via Morego 30, 16163 Genova, Italy; § BiKi Technologies s.r.l., Via XX Settembre 33/10, 1621 Genova, Italy; ∥ CONCEPT Lab, Fondazione Istituto Italiano di Tecnologia, Via Morego 30, 16163 Genova, Italy; ⊥ Dipartimento di Scienze Chimiche, Università di Padova, Via Marzolo 1, 35131 Padova, Italy

## Abstract

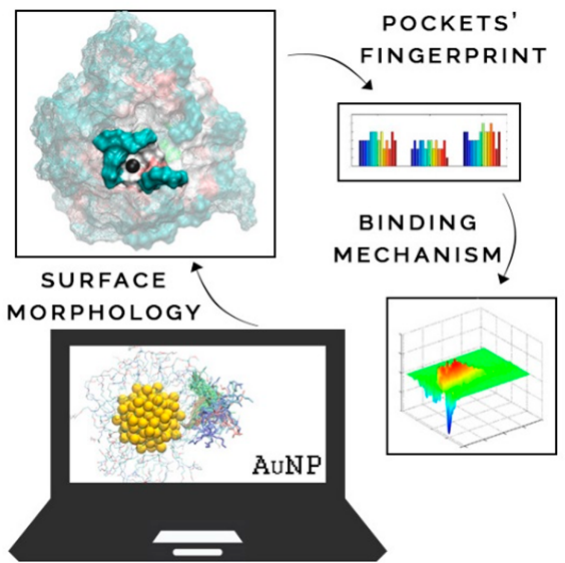

Ligand shell-protected gold nanoparticles can form nanoreceptors that recognize and bind to specific molecules in solution, with numerous potential innovative applications in science and industry. At this stage, the challenge is to rationally design such nanoreceptors to optimize their performance and boost their further development. Toward this aim, we have developed a new computational tool, Nanotron. This allows the analysis of molecular dynamics simulations of ligand shell-protected nanoparticles to define their exact surface morphology and pocket fingerprints of binding cavities in the coating monolayer. Importantly, from dissecting the well-characterized pairing formed by the guest salicylate molecule and specific host nanoreceptors, our work reveals that guest binding at such nanoreceptors occurs via preformed deep pockets in the host. Upon the interaction with the guest, such pockets undergo an induced-fit-like structural optimization for best host–guest fitting. Our findings and methodological advancement will accelerate the rational design of new-generation nanoreceptors.

Over the last three decades, ligand shell-protected metal nanoparticles have been extensively studied to determine and modulate their numerous important properties.^1−8^ Among them, one of the less obvious and still only partially explored is the ability of such functionalized nanoparticles to act as nanoreceptors. Such nanoreceptors have already shown great potential for applications including small-molecule detection in solution (chemosensing),^9−14^ catalysis (nanozymes),^15−20^ and transport of chemical species in biological environments and cells (e.g., drug delivery).^21−25^

The molecular recognition properties of these nanoreceptors are dictated by the chemical structure of the coating ligands, which form self-organized and multivalent binding sites that host the guest species. Such guests are recognized and ultimately positioned into binding sites through noncovalent interactions.^26−31^ The current challenge is now to rationally design such multivalent binding sites so to make them more effective toward the development of intelligent nanoreceptors with superior performance.

Our study is centered on specific and experimentally well-characterized ligand shell-protected nanoparticles such as **1**-AuNP (Chart 1), which is known to recognize salicylate by establishing a combination of hydrophobic and H-bonding interactions inside the guest pocket in the nanoreceptor, as previously shown also via molecular dynamics (MD) simulations.^30,31^ Moreover, we already showed that selectivity for salicylate over the positional isomers 2- and 3-hydroxybenzoate is due to the complementarity between the structure of the binding pockets and that of salicylate.^30^ Indeed, MD-guided specific modifications of the structure of the outer ligand in **1**-AuNP, modified to contain one urea moiety as in **2**-AuNP (Chart 1) to allow for a better pattern of hydrogen bond interactions with the guest, led to more stable and long contacts between **2**-AuNP and the analyte.^31^ Importantly, such MD-derived evidence was confirmed by NMR experiments, which showed a 10-fold affinity improvement for **2**-AuNP over **1**-AuNP. Finally, MD simulations also anticipated that the insertion of a single oxygen atom in the hydrophobic region of the thiol would have made **3**-AuNP (Chart 1) unable to bind the analyte, as then confirmed by experiments.^31^

**Chart 1:**
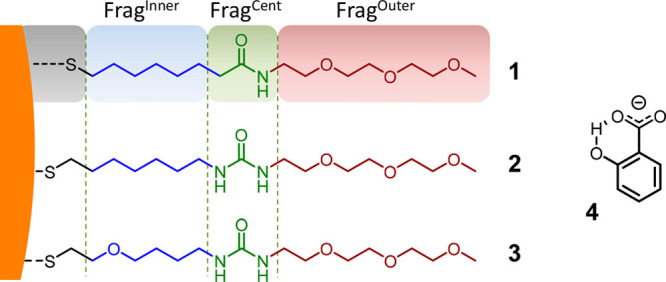
Structure of the Coating Thiols **1**, **2**, **3**, and Analyte **4**, i.e., 2-Hydroxybenzoate (Salicylate)a

a Each coating ligand was divided into fragments: Frag^Inner^ (blue), Frag^Central^ (green), Frag^Outer^ (red). Such ligands are linked to AuNPs of ∼2 nm, represented by the Au_144_(SR)_60_ structure.

Notwithstanding the previously collected information, the binding process of small molecules to the nanoparticle coating ligand shell is still only marginally understood. In particular, the effect of analyte binding on the structure of the monolayer has still to be clarified. In this context, through the use of equilibrium μs-long MD simulations and data analysis on volume-filtered pockets, here we have characterized, at the atomic detail, the pockets formed into the coating monolayer of selected nanoreceptors and achieved a molecular fingerprint of such functional pockets. Our results clarify how the capacity of such nanoreceptors to bind specific small organic analytes depends critically on the physicochemical nature of the thiolate ligands and their topological organization forming the outer functionalized monolayer.

## Methodological Approach

**Figure 1:**
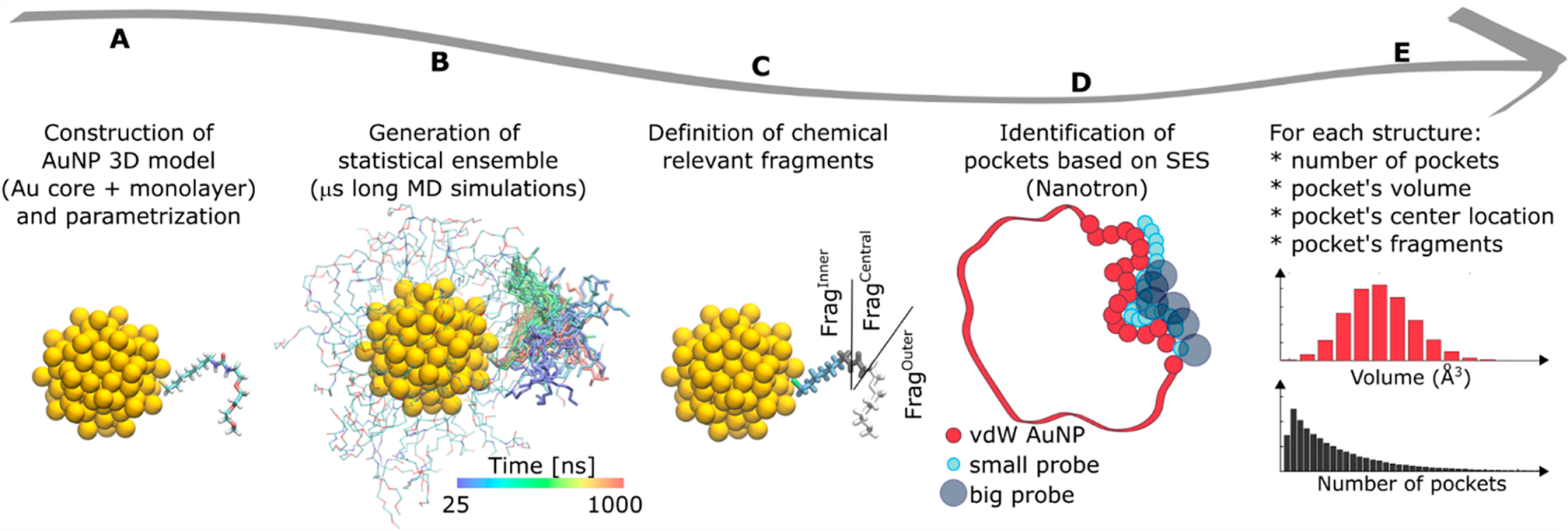
Protocol for the characterization of pockets in AuNPs. (A) The three-dimensional model of the functionalized AuNP is obtained by attaching the coating ligands to the desired gold core. (B) A statistical ensemble of structures of the monolayer self-organization is generated computationally by all-atom MD simulations in explicit solvent. The colored lines show the position of a single ligand at frames taken every 5 ns of simulated time. (C) Each coating ligand is divided into fragments, which are chemical units with unique characteristics. (D) The computational tool Nanotron is used to identify the pockets on the nanoparticle. The pocket volume is obtained as the volumetric difference of two solvent excluded surfaces (SES) of the nanoparticle using two different probe radii (small cyan and big gray rolling spherical probe with a radius of 1.4 and 3 Å, respectively). A candidate pocket is kept if the volume enclosed by the SESs is higher than 100 Å^3^. (E) Nanotron reports, for each structure, the number of pockets and other information about each pocket. This information can be used to better understand the characteristics of the pockets in each AuNP.

We started our study by building three-dimensional (3D, Figure 1A) structures of specific functionalized nanoparticles. In our case, we relied on the Au_144_(SR)_60_ model, which can nowadays be built using the NanoModeler WebServer.^32−35^ Although the Au_144_(SR)_60_ model does not account for the size dispersion of the experimental sample, it is responsible for most of the effect observed in experiments and has been demonstrated to well predict the behavior of ligand shell-protected nanoparticles with average size around 1.6–2.0 nm.^30,31^ Notably, there are only a few experimental structures of gold clusters and AuNPs with core diameters ranging from 0.9 to 2.1 nm (a list of the structures and the respective references can be found in ref (32)). However, the effect of core size modifications on the ability to bind organic molecules in the monolayer was clearly shown by Lucarini and Pasquato for AuNPs coated by ligand-**1** (Chart 1), and it is small in this size range.^28^ The significance of the results acquired is granted by the relevant extension of the simulations of ∼1 μs. Snapshots were analyzed every 50 ps.

We used our models and MD simulations to collect a statistically significant ensemble of AuNPs structures in water. In this way, we could sample conformations of the pockets formed in the outer shell of the AuNP and collect the interactions of each pocket with the analyte, during our MD simulations (Figure 1B).^30,31^ Notably, each coating ligand is formed by three distinct structural fragments (i.e., building blocks, as shown in Chart 1 and Figure 1C): Frag^Inner^ corresponds to the hydrophobic alkyl region in **1**/**2**-AuNP or to the modified alkyl, obtained by inserting a polar oxygen atom into the alkyl chain, in **3**-AuNP; Frag^Central^ is the central characteristic moiety, an amide in **1**-AuNP or one urea in **2**/**3**-AuNP; Frag^Outer^ is the terminal oligo-ethylene glycol (OEG), which was initially inserted to ensure the water solubility of the nanoparticles. This schematic representation of each thiol allowed a detailed analysis of the chemical diversity of the different monolayers’ pockets.

The initial inspection of microsecond-long MD simulations of our AuNP models, already underlines that the molecular surface of the nanoreceptors is pretty rough and rich in cavities of different sizes, which in many cases are just random engulfments of the ligands shell. To analyze such a complex and dynamic surface, we were in need of a new systematic pockets’ detection and classification method capable of handling such systems and MD trajectories. For this reason, we developed Nanotron, which uses the NanoShaper^36^ program to analyze each MD snapshot (Figure 1D and Methods). Notably, NanoShaper^36,37^ and its static pocket detection algorithm—based on the solvent excluded surface concept—were conceived for protein pockets and even used for dynamical analysis of protein pockets (Pocketron),^38,39^ in principle transferable outside the proteins systems. In our case, such an approach for pocket analysis required some further developments because of the complexity of the dynamics and overall mapping of pockets in nanoreceptors. In fact, contrary to proteins where pockets are located in defined regions and are often quite long-lasting in time, if not even permanently preserved in the structure,^40^ the spherical symmetry of nanoparticles makes the appearance and annihilation of pockets a complex phenomenon to monitor and analyze. To address this challenge, here we have used Nanotron to achieve the exact *geolocalization* of pockets in our nanoreceptors, with a newly developed statistical approach that accounts for each MD snapshot independently.

Specifically, the pocket analysis in Nanotron is built on the pocket detection of NanoShaper. This implements the concept of solvent excluded surface (SES), or Connolly–Richards surface, defined as the surface obtained by rolling a spherical probe over the van der Waals surface of the molecular system.^41^ The SES is computed and triangulated following a rigorous procedure.^36^ Pockets are identified by calculating the volumetric difference between the regions enclosed by the SESs, obtained with two different probe radii. The smaller rolling spherical probe has a radius of 1.4 Å, which corresponds to a water molecule’s spherical approximation.

Conversely, the larger rolling spherical probe has a default radius of 3 Å. Here, pockets were identified using such default values, with a minimum cutoff filter set at a volume of 100 Å^3^, approximately the molecular volume of salicylate or the volume of three water molecules in bulk.^38^ Once the pockets were retrieved, we computed and stored the center of mass of each pocket together with its volume. Importantly, we collected the exact atoms forming the pocket’s walls for each pocket, thus estimating the probability that a pocket is formed by a specific thiol and fragments (for more details, see Methods in SI).

In this way, our pocket detection computational tool for the nanoparticle’s surface analyzed through MD simulations provided us with a complete description of the pockets in the nanoparticle’s ligand shell, including the total number of pockets per frame, the volume of each pocket, the IDs of the fragments forming the pocket, and the *xyz* coordinates of the pocket center (Figure 1E). These data were analyzed to quantify the average number of pockets, their *geolocalization* on the nanoparticle’s surface, the physicochemical composition, and how the pockets change upon analyte binding, along the MD simulations. Such information allowed us to define each pocket according to two key features: surface morphology and pocket fingerprints, as discussed in the following paragraphs.

## Surface Morphology

**Figure 2:**
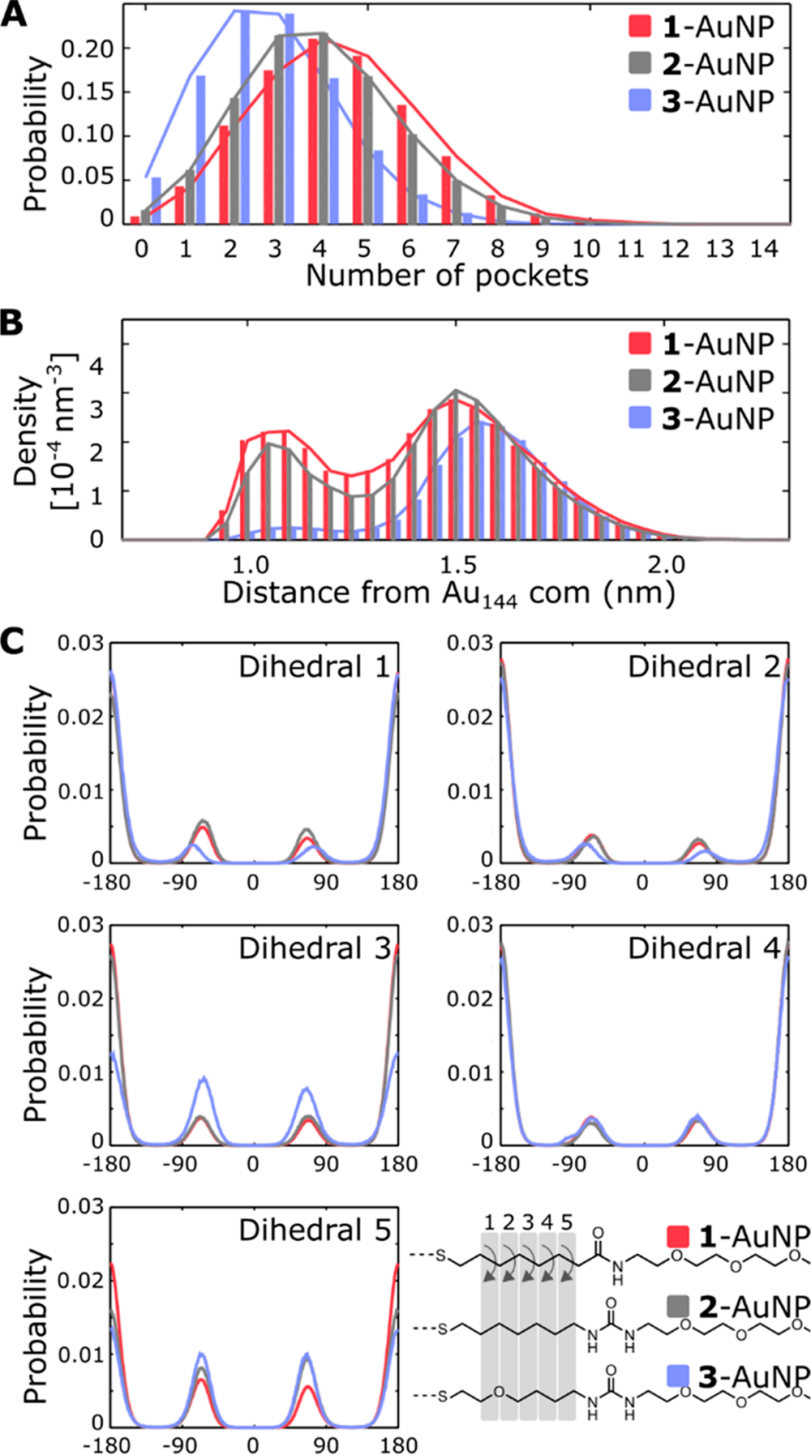
Pockets’ characterization in **1**-, **2**-, and **3**-AuNP simulated alone in explicit solvent. (A) Number of pockets per frame. (B) Pocket’s center distance from the gold core center of mass (com). (C) Distributions of the Frag^Inner^ dihedral angles, which were calculated considering the heavy atoms of the ligands, starting from the first carbon after the sulfur atom (e.g., C_1_–C_2_−C_3_/O_3_–C_4_ for Dihedral 1 until C_5_–C_6_−C_7_–C_8_/N_8_ for Dihedral 5).

Nanotron analysis of μs-long MD simulations in water revealed the simultaneous presence of several pockets in the ligand shell. Namely, 4.4 ± 1.9, 3.9 ± 1.8, and 2.8 ± 1.6 pockets (Figure 2A) were present respectively on **1**-, **2**-, and **3**-AuNPs. However, variations of the total number of pockets are quite broad, spanning from 0 to a maximum of 14 simultaneous pockets. This variation is symptomatic of highly dynamical monolayers, with pockets that frequently open and close in the simulated time scale (Figure S3). Moreover, ∼60% of the pockets have a small size (<150 Å) and only 4% of the pockets have a large volume (>300 Å), as indicated by the skewed distribution of the pocket volume (Figures S4–S6).

The pockets are homogeneously scattered on the AuNP monolayer. The median maximum distance is 2.9, 2.8, and 2.6 nm, for **1**-, **2**-, and **3**-AuNPs, which roughly corresponds to the nanoparticles’ diameter (Figure S4–S6). On the other hand, the distribution of the pockets’ depths is uneven: there is indeed a first group of deep pockets, whose center is located close (∼1.1 nm) to the gold core, and a second one that lies farther, at ∼1.5 nm, from the gold core center (Figure 2B). The relevance of these two populations is different in the three nanoparticles. In the case of **1**- and **2**-AuNP, deeper pockets (distance to the gold core center of mass <1.3 nm) are about 20% of the total, while they sum up to only 4% in the case of **3**-AuNP, which therefore has most pockets on the surface.

The presence of the amide or urea along the coating thiols in the AuNPs has a relevant effect also on the interaction of such thiols among themselves and with the solvent. We found that in **1**-AuNP the amide present in the pockets forms a relatively small (37% with respect to the maximum possible) amount of H-bonds with the amides of other ligands and a large (83%) amount of H-bonds with water molecules. In **2**- and **3**-AuNPs, on the other hand, the presence of the urea group increases both the interligand (more than 70%) and solvent (94%) interactions (Figures S7–S8).

Taken together, the above results suggest that the monolayer coating **3**-AuNP, which shows fewer and shallower pockets, is thus more compact than that of **1**- and **2**-AuNP. A reasonable justification of this behavior is provided by the analysis of the dihedral angles about the bonds of the Frag^Inner^ portion in the three particles (Figure 2C). In **3**-AuNP, in agreement with the conformational preference of ethers, most thiols adopt a folded conformation, with the bond between carbon 4 and 5 found often in the *gauche* conformation. The same bond in **1**- and **2**- AuNPs strongly prefers the *trans* conformation. Hence, the oxygen atom in the Frag^Inner^ of **3**-AuNP allows the ligands to fold and better occupy the curved space around the particle surface. This likely hampers the formation of deep pockets. On the contrary, the preference for the extended conformations of the alkyl chains in **1** and **2** facilitates the formation of such deep pockets.

## Pocket Fingerprints

**Figure 3:**
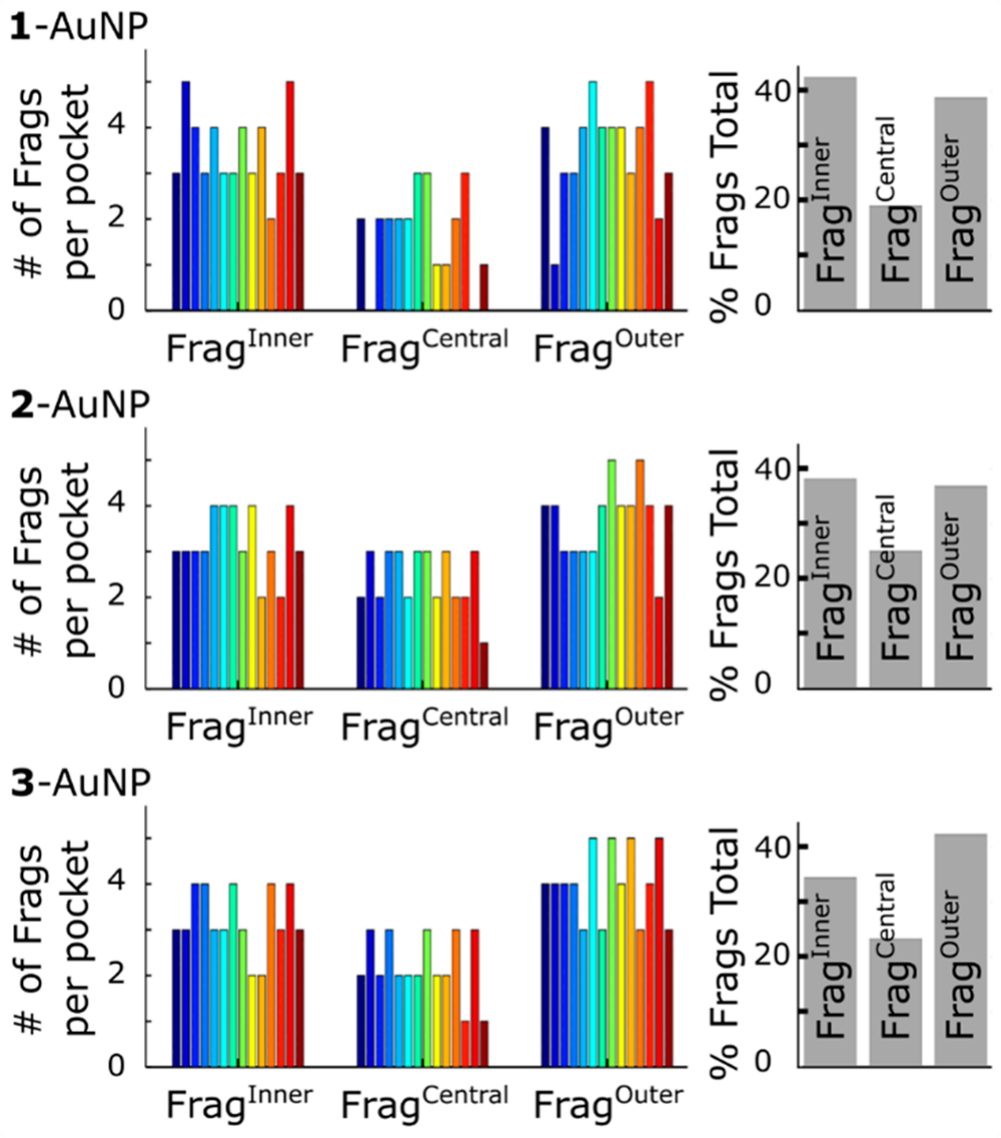
Pockets’ fingerprint for **1**-, **2**-, and **3**-AuNPs. In the left panels, there are the fingerprint of the 14 most populated pockets individually (each color corresponds to a pocket). In the right panels, there is the % of occurrence of the different fragments in all the pockets (gray).

We then moved our attention to the composition, in terms of thiol fragments, of the walls of the pockets in the monolayer. In particular, the number of ligands participating in the formation of a pocket cavity is 6 ± 1 for **1**-AuNP and 8 ± 2 for both **2**- and **3**-AuNP. By breaking the ligands into the three fragments described above (Chart 1), we found that there are about 11 fragments forming each pocket. Hence, in most the cases, the pocket is formed only by a specific fragment of each ligand involved in it. Accordingly, the different fragments are unevenly represented in the pockets’ formation, considering all the AuNPs: 34–42%, 19–24%, 39–43% for Frag^Inner^, Frag^Central^, and Frag^Outer^, respectively (Figure 3). However, pocket composition slightly differs in the three AuNPs. In particular, in **3**-AuNP, Frag^Inner^ is present less frequently (34% vs 38–42% in **1**/**2**-AuNP) and Frag^Outer^ is more present (43% vs 38–39% in **1**/**2**-AuNP). This also agrees with the fact that there are fewer deep pockets formed in **3**-AuNP.

Interestingly, each pocket can be identified by its own fingerprint, which is a feature that univocally defines pockets according to the number of Frag^Inner^, Frag^Central^, and Frag^Outer^ forming it. Thus, there are numerous possible combinations (i.e., fingerprints) of fragments forming a pocket. It is worth underlining that each fingerprint does not correspond to one unique specific pocket but to a set of degenerate pockets with the same number of fragments. Nonetheless, already 14 fingerprints are enough to describe more than 20%, and in some cases almost 30%, of the total pockets (Table S2). The analysis of such recurrent fingerprints confirms that pocket composition does not change sensibly in the three nanoparticles. The majority of the pockets expose a large hydrophobic patch formed by 3–4 fragments. Such pocket cavity also exposes, at the rim, a small number (about 2) of amide/urea groups, and then 3–4 oligo(ethylene glycol) residues (Figure 3). Intriguingly, this suggests that such pockets are formed by a sort of “flower opening mechanism”, through which 3–4 neighboring thiols diverge to open a hydrophobic cavity.

## Analyte Binding

Surface morphology and pocket fingerprints stood out as features capable of differentiating the pockets formed in the ligand shell coating **1**-, **2**-, and **3**-AuNPs. Thus, with this information in our hands, we turned our attention into the changes in the nanoreceptors’ surface when in the presence of salicylate, in solution. Microsecond long MD simulations were collected, each one with 10 substrate molecules (10 salicylate anions and 10 sodium cations) in the solvent box.

**Figure 4:**
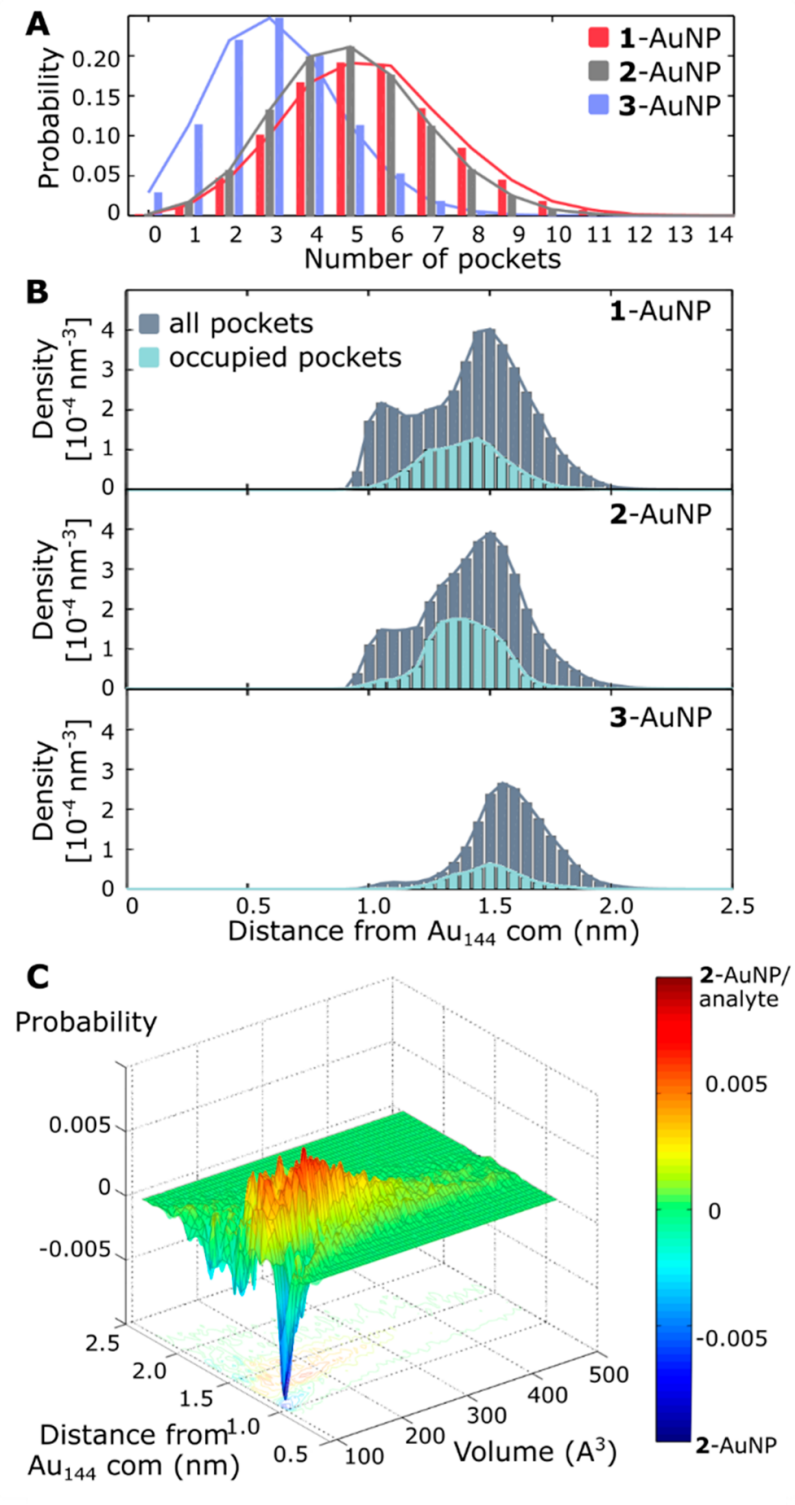
Pockets’ characterization in **1**-, **2**-, and **3**-AuNP simulated with salicylate in explicit solvent. (A) Number of pockets per frame. (B) Localization, from the gold center of mass, of the centers of all the pockets (gray) and of the occupied pockets (cyan). (C) Difference in localization of the pockets and their volume between the monolayer **2**-AuNP in the presence of the analyte (positive values) and the absence of the analyte (negative value).

The first apparent effect of salicylate’s presence was the increase of the average number of pockets, to 5.5 ± 2.0 and 5.0 ± 1.9, in **1**- and **2**-AuNP. On the other hand, in **3**-AuNP, the average number of pockets remained the same, i.e., 3.2 ± 1.6 (Figure 4A). The increase of the number of pockets in **1**- and **2**-AuNPs was accompanied by a change in the distribution of pockets’ volume. We observed a decrease of the number of small pockets and an increase of the larger ones, with the number of pockets larger than 300 Å^3^ that almost doubled.

Also, the distribution of the pocket location into the monolayer (i.e., pockets’ depth) was modified. The two populations individuated in the absence of the analyte were still present but in a different relative amount. In general, the number of deep pockets slightly decreased while that of the shallow ones increased (Figures S4–S7). Besides, a third population of pockets with an intermediate depth (centered at 1.2 nm from the gold core center, Figure 4B) was formed. However, we noted that such changes were modest in **1**-AuNP while quite relevant in the case of **2**-AuNP. On the other hand, they were almost absent in the case of **3**-AuNP, where pockets’ depth before and after the addition of salicylate remained the same.

Crossed analysis of these data revealed that changes in volume and depth distributions were correlated. Indeed, in **1**- and in particular in **2**-AuNP the monolayer experienced, upon addition of the analyte, a substantial decrease of the amount of very deep (1 nm from the gold core center) and small (100 Å^3^) pockets. On the other hand, as shown in Figure 4C, a relevant amount of more voluminous pockets (150–300 Å^3^), located at a higher distance from the gold core center (1.2–1.5 nm), appears in such AuNPs. Eventually, the pockets were classified for the presence of the substrate. In **1**-AuNP, ∼24% of the pockets are occupied by salicylate. The figure rises to ∼33% in **2**-AuNP and decreases to only 10% in the case of **3**-AuNP. Distribution of the occupied pockets, and their relative depth, revealed that they mainly belong to the intermediate depth population, particularly in the case of **2**-AuNP. The structure of occupied pockets presents only minor differences with respect to the empty ones. In all the particles, there was a slight increase of the relevance of Frag^Inner^ exposed in the pockets, from 34 to 42% to 38–43%, and a similar decrease of the amount of Frang^Central^ from 19 to 24% to 17–21%. H-bonding interaction inside the pockets, either interligand or with water molecules, are not sensibly affected by the presence of the analyte. On the other hand, a substantial amount of H-bonds is established with the analyte (15%, 50%, and 35%, respectively for **1**-, **2**-, and **3**-AuNP).

Importantly, our analyses and results show a clear correlation between the number of deep pockets in the coating monolayer and the nanoparticle affinity for the guest. The higher the number of deep pockets, the better the affinity for the analyte in solution, as measured from experiments. However, nanoreceptors’ pockets have, indeed, transient nature and consequently, they cannot be considered structurally defined cavitands. In other words, one would expect that a guest with sufficient affinity should be able to induce the formation of a binding pocket in the monolayer, even if not already present. Nonetheless, **2**- and **3**-AuNP have a very different affinity for salicylate, while these AuNPs behave quite similarly in MD, which is therefore tricky to rationalize.

In this respect, it is tempting to speculate what are the main factors at the basis of the different affinity for salicylate of **2**- and **3**-AuNP, using the evidence from our MD simulations and analyses. Suppose we neglect the contribution of desolvation, which can be similar in all the cases. In that case, we can consider the binding of a guest to the monolayer analogously to the transfer of a solute from vacuum to a solvent. According to theory, energy variation associated with this process can be divided into three contributions: (i) formation of the cavity; (ii) interaction of the guest with the cavity walls; and (iii) reduction of the guest entropy. Comparing our results and ligand shell dynamics for **2**-AuNP and **3**-AuNP in the presence of the analyte, we have shown that the atomic interaction of the analyte with the cavities in such nanoreceptors (via hydrophobic and H-bonding interactions) are virtually identical. Likewise, arguably, it can be inferred that the guest entropy reduction for analyte binding to **2**-AuNP and **3**-AuNP should also be highly similar. Importantly, this would imply that an affinity difference can arise essentially from the energy cost related to the cavity’s opening. From this standpoint, the fact that deep pockets spontaneously form in a relevant amount in **2**-AuNP and not in **3**-AuNP indicates that the cost for their formation is quite different in the two nanoreceptors. Feasibly, this is therefore the most likely reason for their different affinity for salicylate. Measured binding constants (10^3^ M^–1^ for **2**-AuNP vs less than 10 M^–1^ for **3**-AuNP)^31^ allow estimating a lower limit for this cost of ∼11 kJ mol^–1^. The reason for such a difference arises from the different conformational preferences of the ethers with respect to alkenes, which favors a more compact arrangement of the inner portion of the monolayer. On the other hand, our pocket analysis suggests a similar cost for cavity opening in the **1-**AuNP and **2**-AuNP, confirming that the specific chemical structure of the Frag^Inner^ is crucial for analyte recognition and binding. In this case, lower affinity (by ∼5.5 kJ mol^–1^) can be ascribed to the smaller number of H-bonds formed by the amide group with respect to the urea one, as previously proposed. After substrate addition in our MD simulations, the pockets in **1-**AuNP and **2**-AuNP undergo a relevant variation of their shape and position but minimal changes in their composition. Also, the H-bonding network interconnecting the monolayer is not affected. In other words, during this process the cavity enlarges, reducing its depth, but does not change substantially how the fragments form the pocket’s walls. This suggests that the cavity already contains all the features necessary for a complementary interaction with the substrate, that is, H-bond donors and hydrophobic patches. As in an induced-fit mechanism, the cavity only undergoes shape modifications needed to optimize its size and the interactions with the guest. Nicely, this model agrees with the observed selectivity of **1**- and **2**-AuNPs and previous docking calculations that show that only guests with the same interaction pattern of salicylate are recognized by the nanoparticles.^31^

In conclusion, we first developed a new computational tool, Nanotron, to detect and analyze pockets formed during MD trajectories of ligand shell-protected nanoparticles. In this way, we were able to show that guest binding occurs via preformed deep pockets, which upon the interaction with the substrate undergo an induced-fit-like structural evolution and adaptation. Likely, the spontaneous formation of deep pockets avoids adding the costs of monolayer reorganization to the binding process’s energetics. Additionally, we have shown how pocket formation is correlated with the specific chemical structure of the coating molecules. The specific chemical structure correlates with the pocket’s ability to form and effectively recognize the analyte in solution. Eventually, we have found that in the systems studied here, the binding-induced pockets reorganization primarily affects the size and shape of the pocket but not its chemical composition at the cavity walls. This justifies the observed and unexpected selectivity of the studied nanoreceptors. A complete understanding of the characterization and quantification of the relationship between structure and function for such nanoparticles will help the rational design of better nanoreceptors with an affinity toward a target molecule.

## Supporting Information Available

Computational details on MD simulations, computational details on Nanotron (surface characterization, pocket’s identification and classification), analysis for all the investigated systems (PDF)

## References

[ref1] Badia A.; Singh S.; Demers L.; Cuccia L.; Brown G. R.; Lennox R. B. Self-Assembled Monolayers on Gold Nanoparticles. Chem. - Eur. J. 1996, 2, 359–363. 10.1002/chem.19960020318.

[ref2] Saha K.; Agasti S. S.; Kim C.; Li X.; Rotello V. M. Gold Nanoparticles in Chemical and Biological Sensing. Chem. Rev. 2012, 112, 2739–2779. 10.1021/cr2001178. 22295941 PMC4102386

[ref3] Dreaden E. C.; Alkilany A. M.; Huang X.; Murphy C. J.; El-Sayed M. A. The Golden Age: Gold Nanoparticles for Biomedicine. Chem. Soc. Rev. 2012, 41, 2740–2779. 10.1039/C1CS15237H. 22109657 PMC5876014

[ref4] Häkkinen H. The Gold-Sulfur Interface at the Nanoscale. Nat. Chem. 2012, 4, 443–455. 10.1038/nchem.1352. 22614378

[ref5] Pyykkö P. Theoretical Chemistry of Gold. III. Chem. Soc. Rev. 2008, 37, 1967–1997. 10.1039/b708613j. 18762842

[ref6] Mikolajczak D. J.; Berger A. A.; Koksch B. Catalytically Active Peptide-Gold Nanoparticle Conjugates: Prospecting for Artificial Enzymes. Angew. Chem., Int. Ed. 2020, 59, 8776–8785. 10.1002/anie.201908625. PMC7318681 31905254

[ref7] Kotov N. A. Inorganic Nanoparticles as Protein Mimics. Science 2010, 330, 188–189. 10.1126/science.1190094. 20929766

[ref8] Wu M.; Vartanian A. M.; Chong G.; Pandiakumar A. K.; Hamers R. J.; Hernandez R.; Murphy C. J. Solution NMR Analysis of Ligand Environment in Quaternary Ammonium-Terminated Self-Assembled Monolayers on Gold Nanoparticles: The Effect of Surface Curvature and Ligand Structure. J. Am. Chem. Soc. 2019, 141, 4316–4327. 10.1021/jacs.8b11445. 30763078

[ref9] Perrone B.; Springhetti S.; Ramadori F.; Rastrelli F.; Mancin F. ‘NMR Chemosensing’ Using Monolayer-Protected Nanoparticles as Receptors. J. Am. Chem. Soc. 2013, 135, 11768–11771. 10.1021/ja406688a. 23889210

[ref10] Salvia M.; Ramadori F.; Springhetti S.; Diez-Castellnou M.; Perrone B.; Rastrelli F.; Mancin F. Nanoparticle-Assisted NMR Detection of Organic Anions: From Chemosensing to Chromatography. J. Am. Chem. Soc. 2015, 137, 886–892. 10.1021/ja511205e. 25534150

[ref11] Salvia M.-V.; Salassa G.; Rastrelli F.; Mancin F. Turning Supramolecular Receptors into Chemosensors by Nanoparticle-Assisted ‘NMR Chemosensing’. J. Am. Chem. Soc. 2015, 137, 11399–11406. 10.1021/jacs.5b06300. 26313932

[ref12] Gabrielli L.; Rosa-Gastaldo D.; Salvia M.-V.; Springhetti S.; Rastrelli F.; Mancin F. Detection and Identification of Designer Drugs by Nanoparticle-Based NMR Chemosensing. Chem. Sci. 2018, 9, 4777–4784. 10.1039/C8SC01283K. 29910928 PMC5975544

[ref13] De Biasi F.; Mancin F.; Rastrelli F. Nanoparticle-Assisted NMR Spectroscopy: A Chemosensing Perspective. Prog. Nucl. Magn. Reson. Spectrosc. 2020, 117, 70–88. 10.1016/j.pnmrs.2019.12.001. 32471535

[ref14] De Biasi F.; Rosa-Gastaldo D.; Mancin F.; Rastrelli F. Hybrid Nanoreceptors for High Sensitivity Detection of Small Molecules by NMR Chemosensing. Chem. Commun. 2021, 57, 3002 10.1039/D0CC07559K. 33623940

[ref15] Mancin F.; Scrimin P.; Tecilla P. Progress in Artificial Metallonucleases. Chem. Commun. 2012, 48, 5545–5559. 10.1039/c2cc30952a. 22543403

[ref16] Czescik J.; Zamolo S.; Darbre T.; Rigo R.; Sissi C.; Pecina A.; Riccardi L.; De Vivo M.; Mancin F.; Scrimin P. A Gold Nanoparticle Nanonuclease Relying on a Zn(II) Mononuclear Complex. Angew. Chem., Int. Ed. 2021, 60, 1423–1432. 10.1002/anie.202012513. PMC7839518 32985766

[ref17] Chen R.; Neri S.; Prins L. J. Enhanced Catalytic Activity under Non-Equilibrium Conditions. Nat. Nanotechnol. 2020, 15, 868–874. 10.1038/s41565-020-0734-1. 32690887

[ref18] Lou-Franco J.; Das B.; Elliott C.; Cao C. Gold Nanozymes: From Concept to Biomedical Applications. Nano-Micro Lett. 2021, 13, 10 10.1007/s40820-020-00532-z. PMC8187695 34138170

[ref19] Dutta S.; Corni S.; Brancolini G. Molecular Dynamics Simulations of a Catalytic Multivalent Peptide-Nanoparticle Complex. Int. J. Mol. Sci. 2021, 22, 3624 10.3390/ijms22073624. 33807225 PMC8037132

[ref20] Kim M.; Dygas M.; Sobolev Y. I.; Beker W.; Zhuang Q.; Klucznik T.; Ahumada G.; Ahumada J. C.; Grzybowski B. A. On-Nanoparticle Gating Units Render an Ordinary Catalyst Substrate- and Site-Selective. J. Am. Chem. Soc. 2021, 143, 1807–1815. 10.1021/jacs.0c09408. 33471520

[ref21] Yang Y. S. S.; Moynihan K. D.; Bekdemir A.; Dichwalkar T. M.; Noh M. M.; Watson N.; Melo M.; Ingram J.; Suh H.; Ploegh H.; et al. Targeting Small Molecule Drugs to T Cells with Antibody-Directed Cell-Penetrating Gold Nanoparticles. Biomater. Sci. 2019, 7, 113–124. 10.1039/C8BM01208C. PMC6310171 30444251

[ref22] Mottas I.; Bekdemir A.; Cereghetti A.; Spagnuolo L.; Yang Y. S. S.; Müller M.; Irvine D. J.; Stellacci F.; Bourquin C. Amphiphilic Nanoparticle Delivery Enhances the Anticancer Efficacy of a TLR7 Ligand via Local Immune Activation. Biomaterials 2019, 190–191, 111–120. 10.1016/j.biomaterials.2018.10.031. 30415018

[ref23] Ghosh P.; Han G.; De M.; Kim C.; Rotello V. Gold Nanoparticles in Delivery Applications. Adv. Drug Delivery Rev. 2008, 60, 1307–1315. 10.1016/j.addr.2008.03.016. 18555555

[ref24] Chew A. K.; Dallin B. C.; Van Lehn R. C. The Interplay of Ligand Properties and Core Size Dictates the Hydrophobicity of Monolayer-Protected Gold Nanoparticles. ACS Nano 2021, 15, 4534–4545. 10.1021/acsnano.0c08623. 33621066

[ref25] Marson D.; Guida F.; Şologan M.; Boccardo S.; Pengo P.; Perissinotto F.; Iacuzzi V.; Pellizzoni E.; Polizzi S.; Casalis L.; et al. Mixed Fluorinated/Hydrogenated Self-Assembled Monolayer-Protected Gold Nanoparticles: In Silico and In Vitro Behavior. Small 2019, 15, 1900323 10.1002/smll.201900323. 30941901

[ref26] Boal A. K.; Rotello V. M. Fabrication and Self-Optimization of Multivalent Receptors on Nanoparticle Scaffolds. J. Am. Chem. Soc. 2000, 122, 734–735. 10.1021/ja993900s.

[ref27] Daniel M. C. M.; Astruc D. Gold Nanoparticles: Assembly, Supramolecular Chemistry, Quantum-Size Related Properties and Applications toward Biology, Catalysis and Nanotechnology. Chem. Rev. 2004, 104, 293–346. 10.1021/cr030698+. 14719978

[ref28] Lucarini M.; Franchi P.; Pedulli G. F.; Gentilini C.; Polizzi S.; Pengo P.; Scrimin P.; Pasquato L. Effect of Core Size on the Partition of Organic Solutes in the Monolayer of Water-Soluble Nanoparticles: An ESR Investigation. J. Am. Chem. Soc. 2005, 127, 16384–16385. 10.1021/ja0560534. 16305211

[ref29] Pengo P.; Şologan M.; Pasquato L.; Guida F.; Pacor S.; Tossi A.; Stellacci F.; Marson D.; Boccardo S.; Pricl S.; et al. Gold Nanoparticles with Patterned Surface Monolayers for Nanomedicine: Current Perspectives. Eur. Biophys. J. 2017, 46, 749–771. 10.1007/s00249-017-1250-6. 28865004 PMC5693983

[ref30] Riccardi L.; Gabrielli L.; Sun X.; De Biasi F.; Rastrelli F.; Mancin F.; De Vivo M. Nanoparticle-Based Receptors Mimic Protein-Ligand Recognition. Chem. 2017, 3, 92–109. 10.1016/j.chempr.2017.05.016. 28770257 PMC5521955

[ref31] Sun X.; Riccardi L.; De Biasi F.; Rastrelli F.; De Vivo M.; Mancin F. Molecular-Dynamics-Simulation-Directed Rational Design of Nanoreceptors with Targeted Affinity. Angew. Chem., Int. Ed. 2019, 58, 7702–7707. 10.1002/anie.201902316. 30964595

[ref32] Franco-Ulloa S.; Riccardi L.; Rimembrana F.; Pini M.; De Vivo M. NanoModeler: A Webserver for Molecular Simulations and Engineering of Nanoparticles. J. Chem. Theory Comput. 2019, 15, 2022–2032. 10.1021/acs.jctc.8b01304. 30758952

[ref33] Heinz H.; Vaia R. A.; Farmer B. L.; Naik R. R. Accurate Simulation of Surfaces and Interfaces of Face-Centered Cubic Metals Using 12–6 and 9–6 Lennard-Jones Potentials. J. Phys. Chem. C 2008, 112, 17281–17290. 10.1021/jp801931d.

[ref34] Heikkilä E.; Gurtovenko A. A.; Martinez-Seara H.; Häkkinen H.; Vattulainen I.; Akola J. Atomistic Simulations of Functional Au144(SR)60 Gold Nanoparticles in Aqueous Environment. J. Phys. Chem. C 2012, 116, 9805–9815. 10.1021/jp301094m.

[ref35] Pohjolainen E.; Chen X.; Malola S.; Groenhof G.; Häkkinen H. A Unified AMBER-Compatible Molecular Mechanics Force Field for Thiolate-Protected Gold Nanoclusters. J. Chem. Theory Comput. 2016, 12, 1342–1350. 10.1021/acs.jctc.5b01053. 26845636

[ref36] Decherchi S.; Rocchia W. A General and Robust Ray-Casting-Based Algorithm for Triangulating Surfaces at the Nanoscale. PLoS One 2013, 8, e59744 10.1371/journal.pone.0059744. 23577073 PMC3618509

[ref37] Decherchi S.; Spitaleri A.; Stone J.; Rocchia W. NanoShaper-VMD Interface: Computing and Visualizing Surfaces, Pockets and Channels in Molecular Systems. Bioinformatics 2019, 35, 1241–1243. 10.1093/bioinformatics/bty761. 30169777 PMC6449750

[ref38] La Sala G.; Decherchi S.; De Vivo M.; Rocchia W. Allosteric Communication Networks in Proteins Revealed through Pocket Crosstalk Analysis. ACS Cent. Sci. 2017, 3, 949–960. 10.1021/acscentsci.7b00211. 28979936 PMC5620967

[ref39] Decherchi S.; Bottegoni G.; Spitaleri A.; Rocchia W.; Cavalli A. BiKi Life Sciences: A New Suite for Molecular Dynamics and Related Methods in Drug Discovery. J. Chem. Inf. Model. 2018, 58, 219–224. 10.1021/acs.jcim.7b00680. 29338240

[ref40] Stank A.; Kokh D. B.; Fuller J. C.; Wade R. C. Protein Binding Pocket Dynamics. Acc. Chem. Res. 2016, 49, 809–815. 10.1021/acs.accounts.5b00516. 27110726

[ref41] Richards F. M. Areas, Volumes, Packing, and Protein Structure. Annu. Rev. Biophys. Bioeng. 1977, 6, 151–176. 10.1146/annurev.bb.06.060177.001055. 326146

