# Supplementary material for: Molecular Recognition by Gold Nanoparticle-Based Receptors as Defined through Surface Morphology and Pockets Fingerprint
Source: J Phys Chem Lett. 2021 Jun 10;12(23):5616–22. doi: 10.1021/acs.jpclett.1c01365 (PMC8280747; doi:10.1021/acs.jpclett.1c01365)
Supplement: Supplementary file 1 — jz1c01365_si_001.pdf [file jz1c01365_si_001.pdf]

Supplementary Information

Molecular Recognition by Gold Nanoparticle-Based Receptors as Defined through Surface Morphology and Pockets Fingerprint

Laura Riccardi,[a] Sergio Decherchi,[b,c] Walter Rocchia,[c,d] Giordano Zanoni,[e] Andrea Cavalli,[b,c] Fabrizio Mancin,[e,] Marco De Vivo[a,]**

a) Laboratory of Molecular Modeling & Drug Discovery, Fondazione Istituto Italiano di Tecnologia, Via Morego 30, 16163 Genova, Italy

b) Computational and Chemical Biology, Fondazione Istituto Italiano di Tecnologia, Via Morego 30, 16163 Genova, Italy

c) BiKi Technologies s.r.l., Via XX Settembre 33/10, 1621 Genova, Italy

d) CONCEPT Lab, Fondazione Istituto Italiano di Tecnologia, Via Morego 30, 16163 Genova, Italy

e) Dipartimento di Scienze Chimiche, Università di Padova, Via Marzolo 1, 35131 Padova, Italy

Supplementary Information

SOMMARIO

1. **Structural Model** 3
2. **Molecular Simulations (MD) protocol** 4
3. **Construction of the molecular surface of the nanoparticle and identification of the pockets** 6
4. **Characterization of the pockets in the monolayer** 9

1. STRUCTURAL MODEL

The three-dimensional structure of the simulated AuNPs is based on the Au_{144}-$(SR)_{60}$ model of Lopez-Acevedo,[1] which consists of 144 gold atoms and 60 coating groups grafted to the Au_{144} core via sulfur atoms. In the Lopez-Acevedo model the monolayer consists of methyl groups that we substituted with the coating groups **1**, **2**, and **3** shown in Figure 1 in the main text.

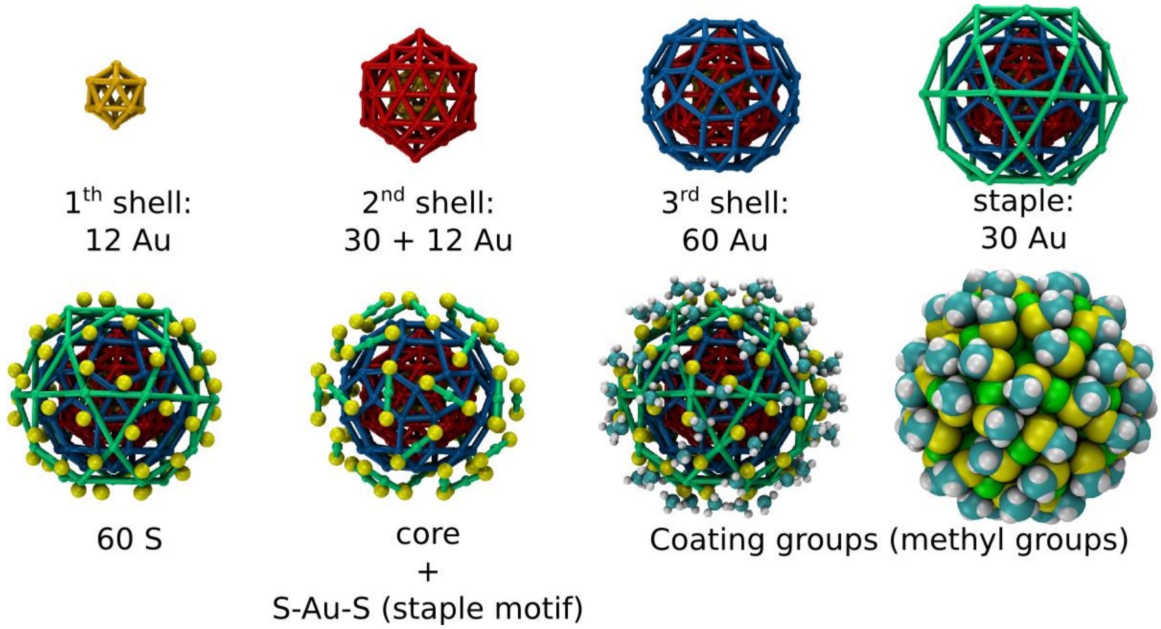

Figure S1. Structure of the Lopez-Acevedo model for Au144-(SR)60, which was used to build up the AuNPs for this study.

To build up the desired monolayer protected AuNP, the heavy atoms of each coating group were placed on the vectors that start from the Au_{144} core and pass through the position of the methyl groups. In this way, the 60 coating groups are extended and farthest away from each other, assuring an unbiased organization of the monolayer in the starting structure.

2. MOLECULAR SIMULATIONS (MD) PROTOCOL

The coating groups as well as the analytes were parametrized with the General Amber Force Fiels (GAFF)[2] and the atomic charges were derived by the RESP[3] fitting procedure, calculated via RedServer (http://q4md-forcefieldtools.org/REDServer-Development), using Gaussian09.[4] In particular, for the coating groups we adopted a force-field topology database building approach, as these coating groups consist of two common building blocks, i.e. the alkyl group close to the sulfur atom and an oligo etilene glycol derivative as a headgroup, and a characteristic central moiety. The notion of molecular-fragment to describe complex structures of repeated units with common regions is used in the derivation of several force-field parametrizations (see Tutorial-IV on the RedServer webpage for more details). This is a combinatorial approach: at first, the coating thiols are divided in building blocks (e.g., alkyl, amide, and OEG region). For each building block, two different molecular conformations were generated with Maestro[5] and atomic charges were calculated as explained above. Then, each coating group was built by combining the proper building blocks. The van der Waals parameters for the gold atoms were taken from the paper of Heinz.[6] To keep the gold/monolayer interface stable (in particular the staple motifs grafted on the surface), further parameters for bonds and angles were added to the force field. The details of the parametrization are described in previous MD simulations of Heikkilä et al.[7] (see Supplementary Information of this reference for all the parameters).

The AuNPs were at first minimized via steepest descent algorithm in vacuum. Then, the simulation box was built to ensure a minimum distance between the AuNPs and the box edges of at least 1 nm and filled up with water (TIP3P model)[8] molecules. A second minimization was applied to relax the solvent molecules around the solute. The system was thermalized up to 300 K coupling the AuNP and the solvent to a V-rescale[9] thermostat (tau_t=0.1) in the canonical ensemble (NVT). Then, we switched to the NPT statistical ensemble, performing 100 ps of MD at 300 K, coupling the system with a Parrinello-Rahman[10] barostat (tau_p=2). After this initial phase the system was ready for productive MD simulations. Production runs were carried out in the NPT (p=1 bar, T=300 K) statistical ensemble. All bonds were constrained with LINCS,[11] allowing to use a time step set of 2 fs. Periodic boundary conditions were applied to the systems in all directions. PME[12] method was used to evaluate long-range electrostatic interactions (pme_order=4, fourier_spacing=0.12), and a cutoff of 10 Å was used to account for the van der Waals interactions. All MD simulations were performed using the GROMACS-4.6.[13,14] For each AuNP in explicit water we performed a >200 ns long MD simulations.
We selected the equilibrated structure of the AuNPs after 100 ns of simulation and used them to build the **1**-AuNP/analyte, **2**-AuNP/analyte, and **3**-AuNP/analyte systems. We built the simulation box as previously and, then, added 10 molecules of analyte (i.e., salicylate) together with 10 Na^+ ions to neutralize the system. The simulations were carried on only in water as this is the solvent

consistent with the NMR chemosensing experiments. Coordinates of the systems were collected every 2 ps, and the analysis performed each 50 ps. After ~25 ns of MD, the Root Mean Square Deviation (RMSD) of the AuNP atoms reached a plateau, ensuring the structural stability of the system, so all analysis refer to the simulations after 25 ns of equilibration.

	**Water**			**Water + salicylate**				
	#AuNP atoms	***#solvent molecules***	***#total atoms***	***#AuNP atoms***	***#analyte molecules***	***#solvent molecules***	***#Na⁺ ions***	***#total atoms***
**1**-AuNP	3204	15034	48306	3204	10	15031	10	48459
**2**-AuNP	3324	16142	51750	3324	10	16222	10	52150
**3**-AuNP	3204	16161	51687	3204	10	16158	10	51838

Table S1. Number of atoms of the investigated AuNPs in water or in water and salicylate.

3. CONSTRUCTION OF THE MOLECULAR SURFACE OF THE NANOPARTICLE AND IDENTIFICATION OF THE POCKETS

Pocket analyser. The pocket tracking algorithm is built around the NanoShaper program, version 0.7.[2] NanoShaper is used as the core engine to identify cavities in the monolayer for each MD frame, as NanoShaper allows to compute all the pockets of a static structure. The pocket detection algorithm of NanoShaper is based on the concept of solvent excluded surface (SES), or Connolly−Richards surface, which is defined as the surface obtained by rolling a spherical probe over the van der Waals surface of the molecular system. The SES in NanoShaper is computed and triangulated following a rigorous procedure.[2] The procedure is composed of the following steps:

- Given atom centers and radii the Regular Delaunay Tethraedralization is computed, let's call it T.
- The complex T is filtered using the Alpha Shape theory to obtain a new complex C which is the skeleton of the surface. This is the equivalent of the reduced surface concept in MSMS.[3]
- From C all the equations of the patches and the corresponding trimming solids can be computed analytically. Let's call this set P.
- Once this representation is available, NanoShaper uses a ray casting technique to sample the surface along regular grid edges. In particular, where possible, the ray (r), surface (S), intersection point p is analytically computed. For the SES a semi-analytical intersection can be computed for torii (re-entrant patches) and fully analytical for spheres (Figure SI).
- Starting from this colored (in/out) grid together with sampled points on the surface, the Marching Cubes algorithm is run to build the triangulated mesh.

Both the ray tracing and Marching Cubes are fully parallelized for shared memory architectures (C++, Boost, Threads).

Upon grid coloring, pockets are identified by calculating the volumetric difference between the regions enclosed by the SESs, obtained with two different probe radii. The smaller rolling spherical probe has a radius of 1.4 Å, which corresponds to the spherical approximation of a water molecule. Conversely, the larger rolling spherical probe has a default radius of 3 Å. The size values can be modified at will. The full procedure is quite complex as care is needed to allow a proper

grid filtering. NanoShaper[2] is free software and it is also available via a GUI and via scripting in the BiKi Life Sciences software suite[4] (www.bikitechnologies.com). The tracking script was written in Groovy, the native scripting language of BiKi Life Sciences. Once the pockets are retrieved via the BiKi API, we compute and store the center of mass of the pocket together with its volume. Moreover, we collect for each pocket the surrounding atoms; thanks to this information we can estimate the probability that a pocket is formed by a specific thiol section.

Additionally, the script builds a 3D grid to estimate the probability to detect a pocket in a specific region of space. To update the grid, the script leverages a feature of NanoShaper, namely its capability to represent a pocket as the union of a set of spheres: given this set of spheres for each pocket it is easy and fast to update the grid accordingly. Finally the grid is saved as a *.dx* file and can be easily visualized by VMD for instance to visualize the regions, chiefly the distance from the nanoparticle center, that exhibit the higher or lower probability regions.

This piece of information can be used to analyze the entire AuNP monolayer, list the identified pockets, and store their calculated volume and the list of contributing Fragments. Finally, for tracking purposes, we removed all the analytes, ions, and water molecules from each trajectory, considering the sole AuNP. The strategy followed here is quite different with respect to the Pocketron[5] which embeds NanoShaper as pocket detection engine with the aim of tracking protein pockets along time in molecular dynamics trajectories. One should observe that Pocketron vanilla application to nanoparticles is not particularly appealing. Indeed, nanoparticles pockets are rather different from their protein counterparts in terms of localization, identity, and kinetics. In the case of proteins, pockets are associated to defined regions and are quite long lasting, when not permanent. In the case of nanoparticles, spherical symmetry makes the appearance and annihilation of pockets a quasi-random phenomenon. Consequently, following the time evolution and correlation of the pockets becomes meaningless.

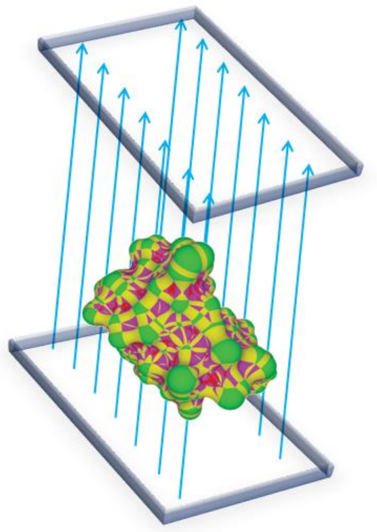

Figure S2. Skin surface with patches as example of the NanoShaper surface sampling strategy. Rays are cast and intersections collected. Once intersections are collected and in/out information is available, the Marching Cubes triangulation takes place. The same strategy is used to sample the SES.

4. CHARACTERIZATION OF THE POCKETS IN THE MONOLAYER

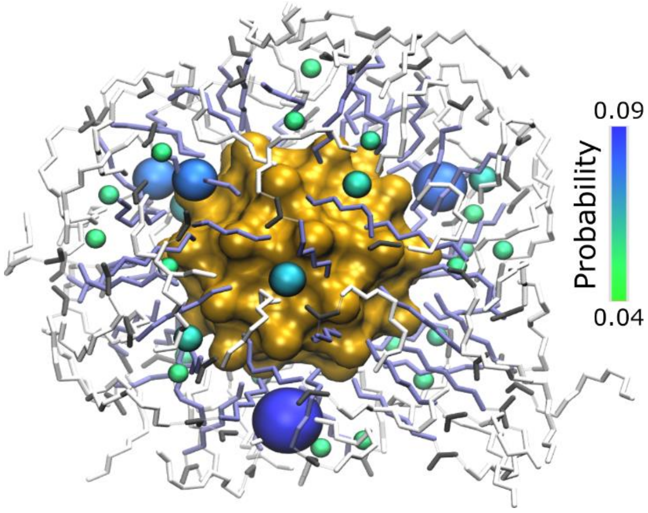

Figure S3. Distribution of the pockets on the 3D structure. The color and size of a sphere reflects the probability to find a pocket in that region of the monolayer. The coating ligands are colored in blue ($Frag^{Inner}$), black ($Frag^{Central}$), and white ($Frag^{Outer}$). Pockets at lower probabilitiesthan 0.04 are not shown for clarity.

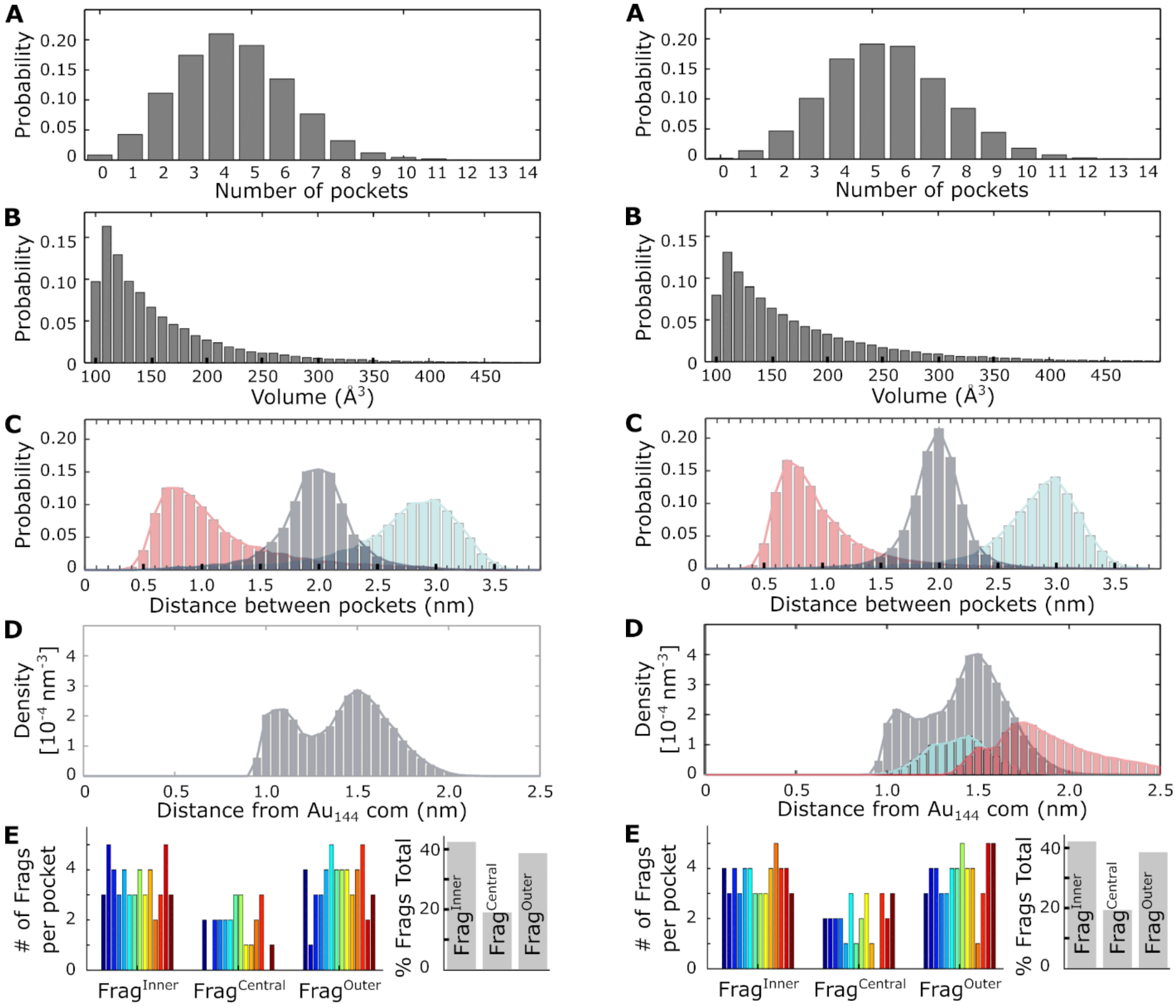

Figure S4. Pockets' characterization in **1**-AuNP (left side) and **1**-AuNP/analyte (right side) systems. **A**) Number of pockets per frame. **B**) Pocket's volume. **C**) Average minimum (red), mean (gray), and maximum (cyan) distances between the pockets. **D**) Localization, from the gold center of mass, of the centers of all the pockets (grey), of the occupied pockets (cyan), and of the salicylate center of mass (red). **E**) Fingerprint of the 14 most populated pockets individually (each color corresponds to a pocket) and all the pockets (gray).

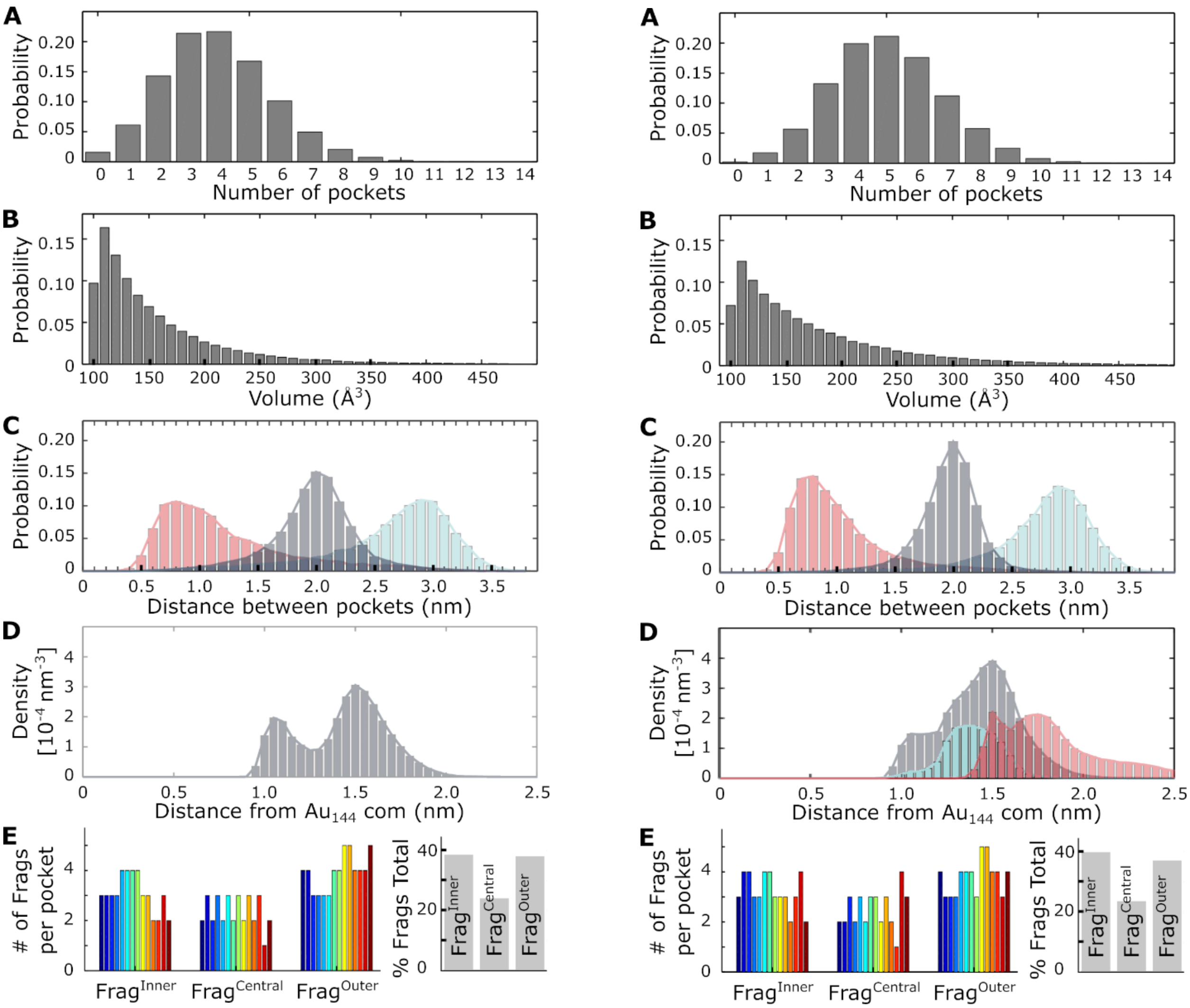

Figure S5. Pockets' characterization in **2**-AuNP (left side) and **2**-AuNP/analyte (right side) systems. **A**) Number of pockets per frame. **B**) Pocket's volume. **C**) Average minimum (red), mean (gray), and maximum (cyan) distances between the pockets. **D**) Localization, from the gold center of mass, of the centers of all the pockets (grey), of the occupied pockets (cyan), and of the salicylate center of mass (red). **E**) Fingerprint of the 14 most populated pockets individually (each color corresponds to a pocket) and all the pockets (gray).

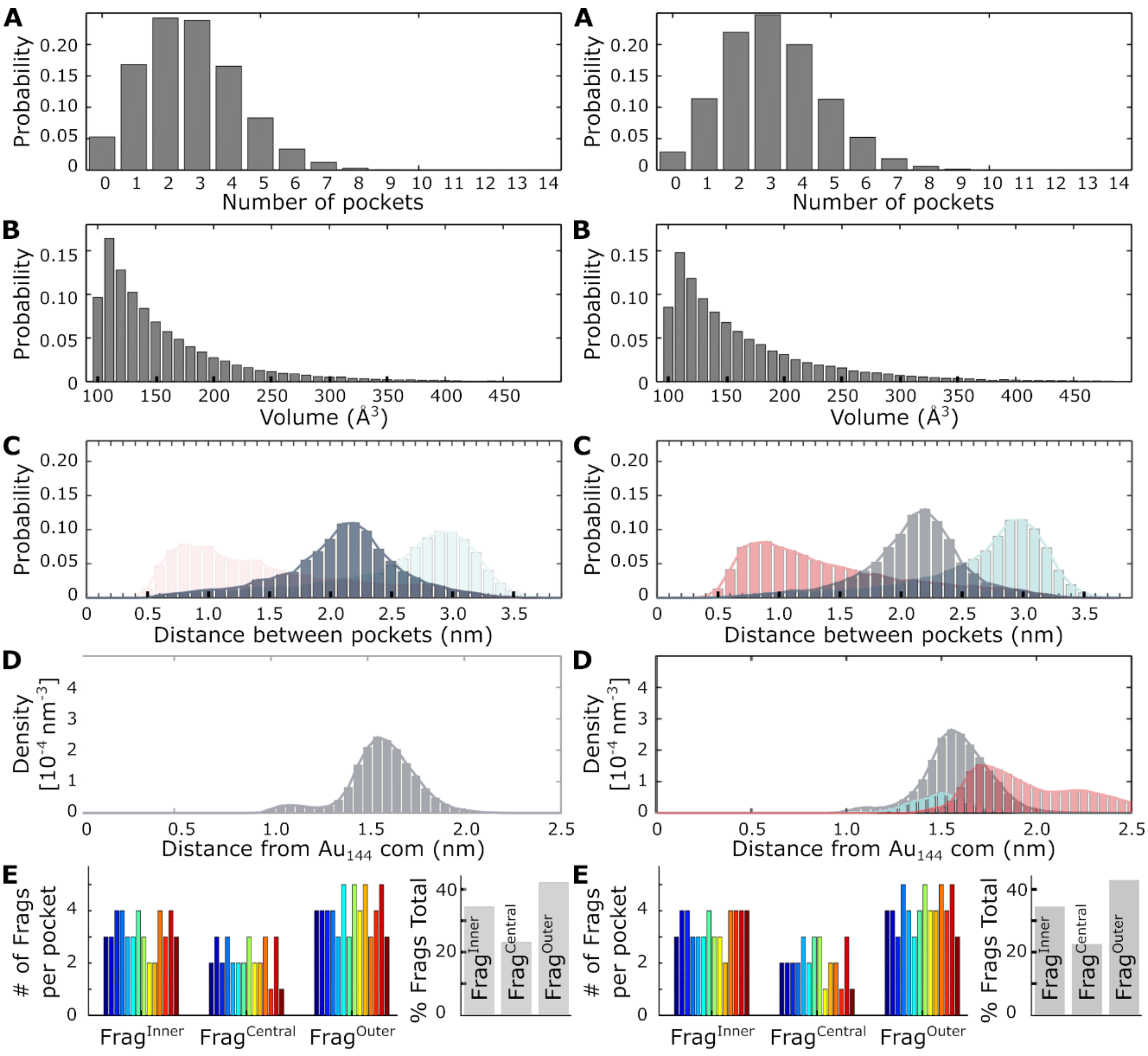

Figure S6. Pockets' characterization in **3**-AuNP (left side) and **3**-AuNP/analyte (right side) systems. **A**) Number of pockets per frame. **B**) Pocket's volume. **C**) Average minimum (red), mean (gray), and maximum (cyan) distances between the pockets. **D**) Localization, from the gold center of mass, of the centers of all the pockets (grey), of the occupied pockets (cyan), and of the salicylate center of mass (red). **E**) Fingerprint of the 14 most populated pockets individually (each color corresponds to a pocket) and all the pockets (gray).

	TOTAL				BOUND		
	% Frag^{Inner}-Frag^{Central}-Frag^{Outer}	14 most populated pockets represent:	Number fragments	Number ligands	% Frag^{Inner}-Frag^{Central}-Frag^{Outer}	14 most populated pockets represent:	% of occupied pockets
**1**-AuNP	42/19/39	26%	9.7±2.7	5.9±1.4			
**1**-AuNP/an	42/19/39	24%	10.1±2.8	6.1±1.5	43/17/39	22%	24%
**2**-AuNP	38/24/38	25%	9.9±2.5	8.3±2.0			
**2**-AuNP/an	40/23/37	20%	10.4±2.8	8.8±2.4	43/21/35	17%	33%
**3**-AuNP	34/23/43	29%	10.2±2.6	8.2±2.0			
**3**-AuNP/an	34/23/43	28%	10.4±2.7	8.4±2.1	38/19/43	30%	19%

Table S2. Percentage for each fragment in the composition of the total pockets or only of the bound ones. Data refer to the simulations of **1**-AuNP, **2**-AuNP, and **3**-AuNP simulated alone or with the analyte (AuNP/an).

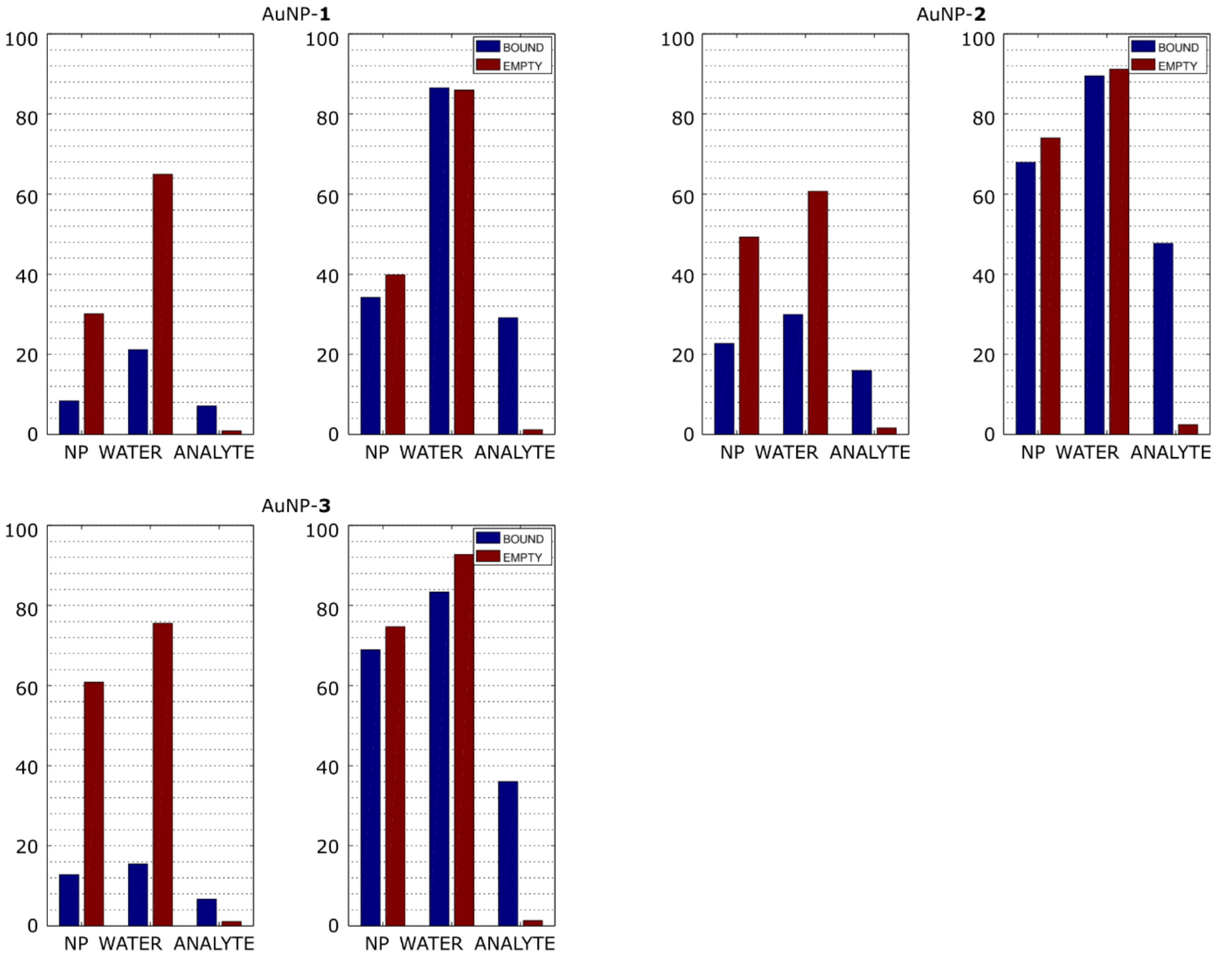

Figure S7. H-bonds between the $Frag^{Central}$ present in a pocket and other $Frag^{Central}$, water molecules, or analytes, for **1**-AuNP, **2**-AuNP, and **3**-AuNP. Blu bars refer to the $Frag^{Central}$ present in bound pockets, while red bars to $Frag^{Central}$ present in empty pockets. The data refers to the simulations of the systems in the presence of the analyte. For each system, in the left plot the histograms are normalized by the total number of pockets and the right plot by the number of bound or empty pockets, for the clue and red bars respectively.

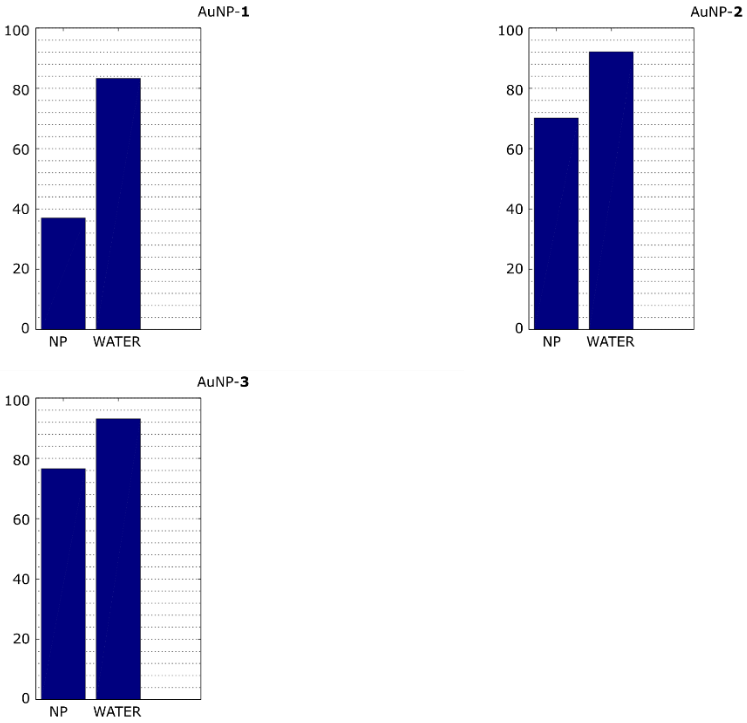

Figure S8. H-bonds between the Frag^{Central} present in a pocket and other Frag^{Central} and water molecules, for **1**-AuNP, **2**-AuNP, and **3**-AuNP. The data refers to the simulations of the systems without the analyte. For each system, the histograms are normalized by the total number of pockets.

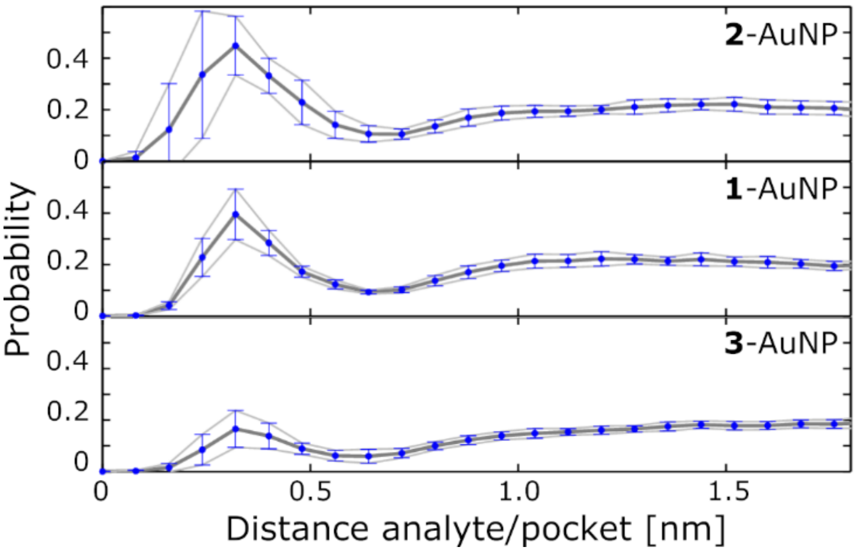

Figure S9. Minimum distance between the center of mass of the analytes and the center of the closest pocket for **2**-AuNP, **1**-AuNP, and **3**-AuNP. The standard deviation was calculated over the 10 analytes present in the MD simulations.

References

(1) Lopez-Acevedo, O.; Akola, J.; Whetten, R. L.; Grönbeck, H.; Häkkinen, H. Structure and Bonding in the Ubiquitous Icosahedral Metallic Gold Cluster Au144(SR)60. *J. Phys. Chem. C* **2009**, *113*, 5035–5038.

(2) Decherchi, S.; Rocchia, W. A General and Robust Ray-Casting-Based Algorithm for Triangulating Surfaces at the Nanoscale. *PLoS One* **2013**, *8*, e59744.

(3) Sanner, M. F.; Olson, A. J.; Spehner, J.-C. Reduced Surface: An Efficient Way to Compute Molecular Surfaces. *Biopolymers* **1996**, *38*, 305–320.

(4) Decherchi, S.; Bottegoni, G.; Spitaleri, A.; Rocchia, W.; Cavalli, A. BiKi Life Sciences: A New Suite for Molecular Dynamics and Related Methods in Drug Discovery. *J. Chem. Inf. Model.* **2018**, *58*, 219–224.

(5) La Sala, G.; Decherchi, S.; De Vivo, M.; Rocchia, W. Allosteric Communication Networks in Proteins Revealed through Pocket Crosstalk Analysis. *ACS Cent. Sci.* **2017**, *3*, 949–960.
